# Treatment of Intracranial Tumors With Stereotactic Radiosurgery: Short-Term Results From Cuba

**DOI:** 10.7759/cureus.29955

**Published:** 2022-10-05

**Authors:** Amaya Alvarez Aquino, Manuel de Jesus Encarnacion Ramirez, Ismail Bozkurt, José Antonio Asprilla González, Evgeniy Goncharov, Ana D Caballero, Renat Nurmukhametov, Nicola Montemurro, Bipin Chaurasia

**Affiliations:** 1 Neurosurgery, International Center of Neurological Restoration (CIREN), Havana, CUB; 2 Neurological Surgery, Peoples' Friendship University of Russia, Moscow, RUS; 3 Neurosurgery, Cankiri State Hospital, Çankırı, TUR; 4 Neurosurgery, Sheba Medical Center, Tel HaShomer Hospital, Tel Aviv, ISR; 5 Traumatology and Orthopedics, Central Clinical Hospital of the Russian Academy of Sciences, Moscow, RUS; 6 Neurosurgery, Tyumen State University, Moscow, RUS; 7 Neurosurgery, Peoples' Friendship University of Russia, Moscow, RUS; 8 Neurosurgery, Azienda Ospedaliero Universitaria Pisana (AOUP), Pisa, ITA; 9 Neurosurgery, Bhawani Hospital and Research Center, Birgunj, NPL

**Keywords:** meningioma, cranial tumors, brain tumors cns tumors, linac based radiosurgery, stereotactic radiosurgery srs

## Abstract

Background

Although international publications on radiosurgery have increased exponentially, reports of heterogeneous series treated with linear accelerator (LINAC) are scarce. Since most intracranial tumors are irregular in size and not spherical, LINACs (Elekta Precise®, Elekta AB, Sweden), fitted with a multi-leaf collimator, allow for precise stereotactic radiosurgery for the entire tumor.

Aim

To evaluate the effects of LINAC on an outpatient basis with patients diagnosed with various intracranial malignancies.

Methodology

A retrospective observational study of a series of cases of patients with intracranial lesions treated at the Institute of Oncology and Radiobiology using LINAC was carried out from October 2019 to May 2021 to evaluate the therapeutic results of radiosurgery in patients with intracranial tumors.

Results

A total of 22 lesions in 20 patients were treated with LINAC. The average age of the patients was 49.7, and the male-female ratio was 1:2. The cases consisted were mostly vestibular schwannoma (7 lesions), metastases from breast cancer (3 lesions), and tuberculum sellae meningioma (2 lesions). The prescription dose covered 99% of the planning target volume in 16 lesions (72.7%) and 100% in six lesions (27.3%) (prescription volume). In meningiomas and schwannomas, doses between 12 and 14 Gy were used, in plasmacytoma 13 Gy, in pilocytic astrocytoma 14 Gy, in cavernoma 15 Gy, in breast cancer metastasis between 18 and 20 Gy, and in lung cancer metastasis 22 Gy. When evaluating local control, 11 patients exhibited stable findings at the six-month control while 10 had partial regression, and a single patient had total regression. Minor complications such as perilesional edema, facial paresthesia, facial paralysis, and transient alopecia were observed in eight of the patients.

Conclusions

Patients with extra-axial, low-grade malignancy, and posterior fossa lesions were predominant in the studied population. Radiosurgery treatment is associated with good local control of the treated lesions. Complications are infrequent, mild, and predominated by perilesional edema.

## Introduction

Stereotactic radiosurgery (SRS) is an interdisciplinary procedure that involves the use of high-resolution anatomical images and specialized equipment supported by high-definition stereoscopic images, combining the principles of stereotaxy with intense focal radiation techniques. The aim of SRS is to localize the irradiation to the tumor tissue while preserving healthy cells [1]. This technique is provided in a single session. Although when it is performed between two and five sessions, it is called hypofractionated stereotactic radiotherapy [2].

The goal of the SRS is to deliver a large obliterative dose to the tumor with great precision and compliance to minimize the dose to surrounding healthy tissues. To meet this objective, there are a series of basic requirements that must be met, such as exact localization, mechanical precision, exact and optimal distribution of the dose, and patient safety [3].

SRS can be used as an alternative or an adjunct to surgical intervention for intracranial lesions, including cerebral vascular malformations and malignant and benign neoplasm. It is also used in patients in whom surgery is not indicated or in residual post-surgical lesions [4]. One of its advantages over surgery is that complications derived from the surgical procedure are avoided, and it is performed on an outpatient basis, thus reducing costs for the patient, institutions, and society [5]. Although it is a less invasive procedure, it is not exempt from complications such as radio-necrosis or the appearance of radio-induced tumors in the long term [6-7].

In recent years, international publications on SRS have increased exponentially. The development of more efficient SRS equipment, high-resolution images for planning, and the international experiences gained have consolidated this treatment modality as a recommended option for many patients and have expanded its indications [4]. This fact makes the analysis of a series of cases treated with SRS a topical issue. SRS has evolved as a well-delivering technology that allows for more precise radiotherapy to the lesion while preserving healthy tissue. More recently, the multi-sheet microcollimator was developed in which sheets of tungsten are used to form the field. The use of a multilayer microcollimator substantially improves dose distribution for irregularly formed tumors while excluding healthy structures, with an additional advantage of a single isocenter, resulting in greater dose homogeneity within the target volume. Furthermore, the ability of multilayer collimators to move during SRS opens up the possibility for the clinical use of modern techniques such as shaped arcs, stereotactically modulated intensity, and, more recently, volumetric modulated arc therapy (VMAT) [8-11]. This retrospective single-center study aimed to evaluate the results of the treatment of various intracranial malignancies by a linear accelerator (LINAC) with a cone collimation system and Elekta Precise® (Elekta AB, Sweden) equipped with a micro-multileaf collimator on various intracranial malignancies.

## Materials and methods

Ethical aspects

A retrospective, observational case series study of patients with intracranial lesions treated at the Institute of Oncology and Radiobiology (INOR) using SRS was conducted from October 2019 to May 2021. The research was carried out according to the principles established in the Declaration of Helsinki, seventh revision (Brazil, 2013) [12] and was approved by the Radiotherapy Service, the Scientific Council and the Medical Ethics Committee of INOR, and the International Center of Neurologic Restoration (CIREN) (06/2021). Patients signed the Act of Consent Informed before proceeding according to the treatment protocol with SRS of the INOR. The confidentiality of patient data was respected.

Inclusion criteria

Patients with single or multiple intracranial lesions who were treated with SRS, with age ≥19 years, and with a minimum follow-up of six months were included.

Exclusion criteria

Patients who were treated with more than one session (fractionation) and were treated in the INOR but by an SRS group from another institution were excluded.

Variables

Patient demographic data, clinical presentation, pre-diagnosis, histological diagnosis, previous treatment, and lesion localization, dimension, and margins were collected. With regards to the treatment, the Paddick Compliance Index [13], prescription dosage and volume, number of arches, type of collimation system, and treatment duration (time elapsed from the placement of the stereotactic frame to its removal) were collected. As a measure of effectiveness, local control of the treated lesions and radiological changes were evaluated. To assess safety, the type and severity of complications were taken into account.

Data collection

The data were obtained from the SRS treatment records of the Radiotherapy Service of the INOR, video imaging studies, and patients. For the statistical processing of the information collected, a database was created in Microsoft Excel and processed using the statistical package STATA S.E. version 11.0 (Stata, USA).

Statistical analysis

Absolute and relative frequencies are used as a summary measure of categorical variables. In the case of quantitative variables, the mean, SD, and median were used. As a measure of the effectiveness of the treatment, local control was estimated at six months, and the frequency and type of complications were measured as a safety measure. Finally, Fisher's exact test was used to correlate qualitative variables.

Procedures

The equipment for the SRS consisted of a LINAC Elekta Precise® (Elekta AB, Sweden), to which a microcollimator multilayer apex was attached. The Leksell stereotactic framework (Elekta AB, Sweden) was used and planned with the Monaco planning system (Elekta AB, Sweden). The procedure was performed on an outpatient basis in the radiotherapy room of the INOR. The stereotactic frame was placed under local anesthesia. After the contrast-enhanced (C+) CT/MRI planning, the patient was transferred to a designated place until the planning process was concluded. To place stereotactic coordinates to the voxels, the fiducials are located in each cut. Then, using image fusion software, the two initial images (CT and MRI) were corrected, and the fusion image was generated. On the C+ stereotactic CT, the targets and the risk organs to be protected are delineated. CT and MRI fusion imaging were used as a guide and test. The optimal directions and amplitudes of the typical radiation beam input arcs were then arranged, defining the specific details depending on the characteristics of each patient. Finally, the neurosurgeon and the radiation therapist defined in consensus the dose (Dmax: 12-15 Gy/6-10 cm3) that should be reached in the plan, and this value was prescribed. Using the Monaco program, the corresponding calculations were carried out, and the three-dimensional dose distribution achieved was evaluated using the available visualization tools and the use of dose and volume histograms and Paddick conformity indices to ensure both good target coverage and protection of risk organs. When dose planning was completed, the patient was placed on the LINAC table, and the radiation was applied.

Dosimetry

A prescription was used according to the SRS protocol of the National Radiosurgery Group, taking into account the diagnosis and size. Previous holo-cranial radiotherapy (HRT), the neurological status of the patient, associated edema, and proximity to eloquent areas were considered before the final dosage decision. The number of lesions was only decisive when they were extremely close, and the dose distribution study indicated excessive focal elevations (hot spots).

## Results

Twenty-two lesions were treated with SRS in 20 patients. Two patients had two lesions each (a patient with neurofibromatosis type 2 with a residual vestibular schwannoma and a residual torcular meningioma, and one with two cerebellar breast cancer metastases). The average age of the patients was 49.7 years (range 28-71). Twelve of the patients were women (60%).

The most frequent clinical presentation was headache and hearing loss with eight patients each (40%), followed by ataxia in three patients (15%) and one patient (5%) who presented with seizures.

Vestibular schwannoma (7 lesions), breast cancer metastases (3 lesions), and tuberculum sellae meningioma (2 lesions) were the most common pathologies. Half of the patients had a histologically confirmed diagnosis because they received surgical treatment before SRS (residual lesions) (Table 1). There were 17 benign and five malignant lesions. Fifteen of the lesions were extra-axial (68.2%), and seven were intra-axial (31.8%). The treated lesions had an average volume of 5.2 ml^3^ (range: 0.4-28.2 ml^3^). Twelve lesions received no previous treatment, while nine were treated with surgery and two with HRT.

**Table 1:** Distribution of lesions.

Presumptive diagnosis	Confirmation by biopsy		Total
	Yes	No	
Vestibular schwannoma	3	4	7
Breast cancer metastases	-	3	3
Tuberculum sellae meningioma	2	-	2
Jugular foramen meningioma	1	-	1
Cerebellar pilocytic astrocytoma	1	-	1
Pineocytoma	1	-	1
Pontocerebellar angle meningioma	-	1	1
Cavernoma (insula)	-	1	1
Parasagittal meningioma	-	1	1
Clival plasmacytoma	1	-	1
Clinoid meningioma	1	-	1
Lung cancer metastasis	-	1	1
Intraventricular meningioma	-	1	1
Total	10	12	22

The cone collimation system was used in 17 lesions (77.3%) using cones of 12.5, 20, 25, and 30 mm depending on the diameter of the tumor; while in five lesions (22.7%), the Apex system (multilayer microcollimator) (Elekta, United Kingdom) was used to treat more irregular lesions.

The prescription dose covered 99% of the planning target volume (PTV) in 16 lesions (72.7%) and 100% in six lesions (27.3%) (prescription volume). In meningiomas and schwannomas, doses between 12 and 14 Gy were used, 13 Gy for plasmacytoma, 14 Gy for pilocytic astrocytoma, 15 Gy for cavernoma, between 18 and 20 Gy for breast cancer metastasis, and 22 Gy for lung cancer metastasis. 

To protect the optic pathway, no margins were used in two patients with tuberculum sellae meningioma. In the remaining 20 patients, a margin of 1 mm was included. The average Paddick compliance index was 0.68 (0.10-0.79). Five to fourteen treatment arches were used: five arches in three patients, six arches in eight patients, seven arches in five patients, and 10 and 14 arches in a single patient, respectively. The average treatment time was 7 hours and 30 minutes (range: 6-10 hours).

Determination of local control of treated lesions

**Figure 1:**
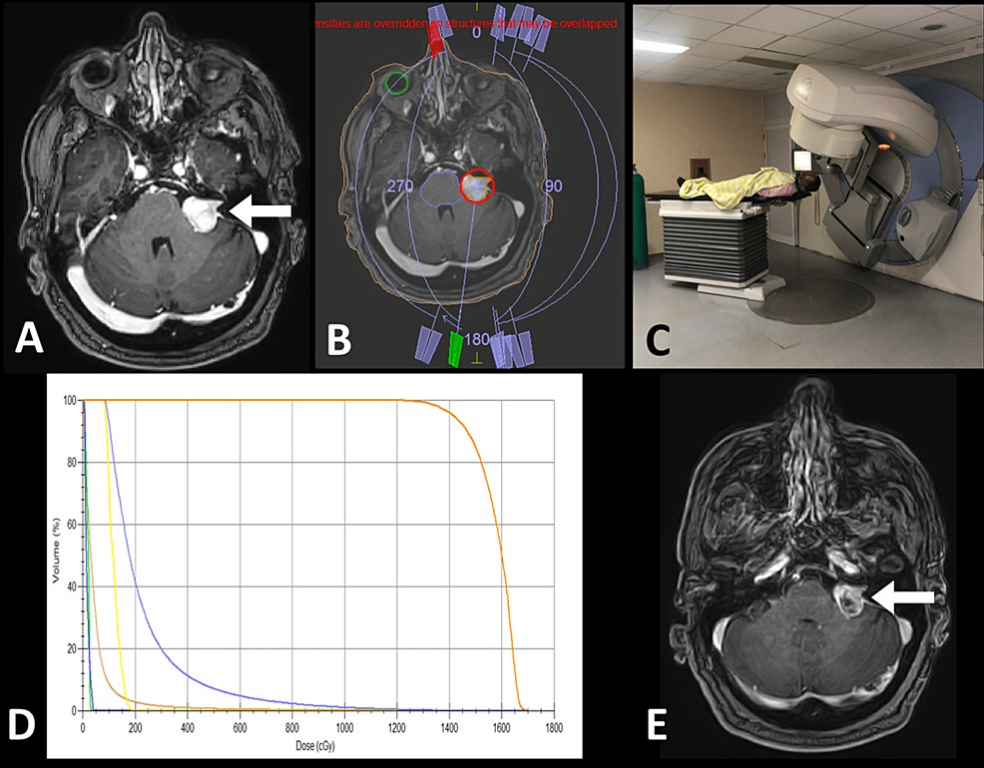
Patient with vestibular schwannoma. A: MRI planning. Arrow: Lesion at the left cerebellopontine angle. B: The planning CT image is observed with the treatment arches. C: Positioning of the patient in the linear accelerator (LINAC). D: Dose/volume histogram. E: MRI C+ at six months of treatment where partial regression and necrotic center are observed (arrow).

**Figure 2:**
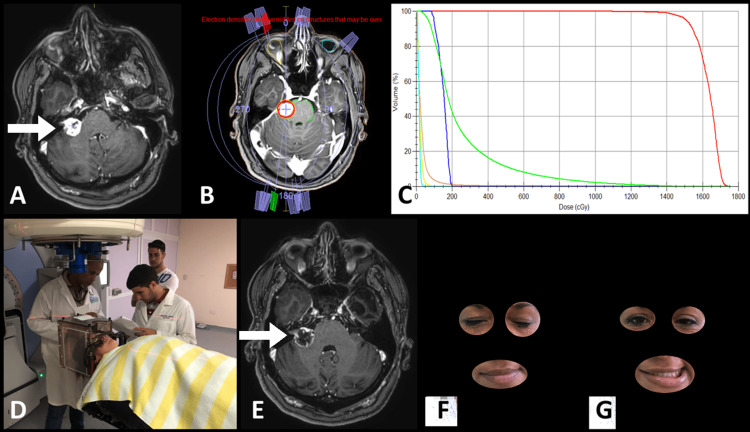
Patient with meningioma of the pontocerebellar angle. A: MRI planning (Arrow: lesion). B: The planning MR image is observed with the treatment arches. C: Histogram dose/volume. D: Positioning in linear accelerator (LINAC). The cone coupled to the accelerator is observed: E: MRI C+ at 12 months of treatment, where partial regression and necrotic center are observed (arrow). F and G: Photographs of the patient (with consent) where facial paresis is observed one year after treatment.

**Figure 3:**
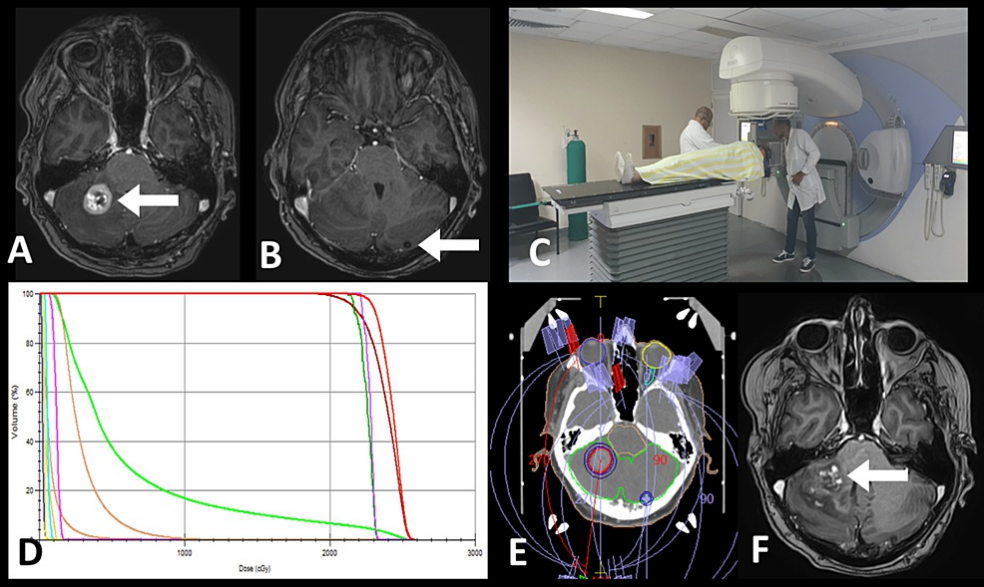
Patient with multiple breast cancer metastases. A: MRI planning where the largest lesion (arrow) is observed at the level of the right cerebellar hemisphere. B: The smallest hypo-intense lesion (arrow) is observed at the level of the right cerebellar hemisphere. C: Positioning of the patient in the linear accelerator (LINAC). The planning CT image is observed with the treatment arches. C: Positioning of the patient in the LINAC. D: Dose/volume histogram. E: Planning CT. F: MRI C+ at nine months of treatment where partial regression and necrotic center, as well as slight perilesional edema, are observed (arrow).

**Figure 4:**
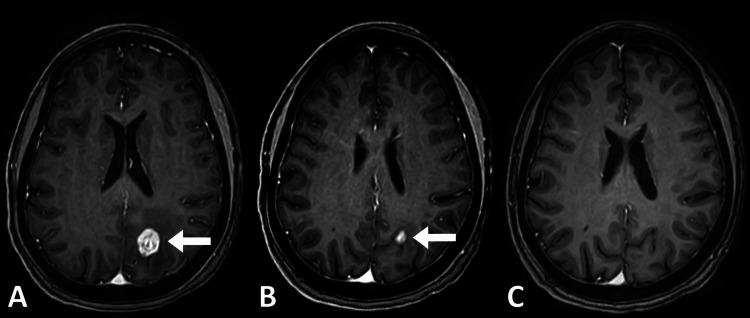
Patient treated with SRS for breast cancer metastasis. MRI C+ before (A), at six months (B), and at 12 months of treatment (C). Total regression of the lesion is observed. (Arrows: lesion). SRS: Stereotactic radiosurgery.

When determining local control at six months, in 11 patients, the disease remained stable (50.0%). In 10, there was a partial regression (45.5%), while in the patient treated for plasmacytoma, a total regression was observed (4.5%). Stable disease was observed mainly in benign lesions such as schwannomas and meningiomas, while partial or total regression was observed mainly in malignant lesions (p=0.039) (Table 2 and Figures 1-4).

**Table 2:** Local control at six months according to histological diagnosis. * Fisher's exact test. CI: 95%.

Histological diagnosis				Total	P-value*
	Total regression	Partial regression	Stable disease		
Schwannoma	-	2	5	7	0.039
Meningioma	-	2	5	7	
Breast cancer metastases	-	3	-	3	
Pineocytoma	-	1	-	1	
Cavernoma	-	1	-	1	
Pilocytic astrocytoma	-	-	1	1	
Lung cancer metastasis	-	1	-	1	
Clival plasmacytoma	1	-	-	1	
Total	1	10	11	22	

Identification of associated complications

Complications were observed in eight patients (40%). Among these, three patients presented with perilesional edema (cavernoma, breast metastases and pilocytic astrocytoma), two patients presented with facial paresthesias (vestibular schwannoma and one pontocerebellar angle meningioma), two patients presented with facial paralysis (vestibular schwannoma), and one patient presented with transient alopecia (parasagittal meningioma). All complications were classified as minor and corresponded to grades I and II according to the Clavien-Dindo Classification [14].

## Discussion

Indications of SRS

SRS is currently used to treat patients with a variety of processes including cerebral vascular malformations (arteriovenous malformations or AVMs [15] and cavernous angiomas [16]), benign tumors (meningiomas [17-18], vestibular schwannoma [19], pituitary adenoma [20-21], glomus jugulare tumors [22], low-grade glioma [23]), and malignant tumors (metastasis [24], chordoma [25], high-grade glioma [26]). Other less common indications are Cushing's disease [27-28], epilepsy [29], trigeminal neuralgia [30], and movement disorders [31].

Most of the series of patients treated with SRS are based on reports of specific lesions. Publications of heterogeneous patient series are observed less frequently [4]. This heterogeneous series had a predominance of the female sex, which can be explained by the fact that breast metastasis (the most frequent malignant tumor of those irradiated in this study) and meningioma predominate in women. Regarding the etiological distribution, a higher incidence of vestibular schwannoma, metastasis, and meningioma is observed. These data coincide with previous publications because these diagnoses are the first indications of SRS [4].

SRS, being a high-tech treatment, represents a challenge in underdeveloped countries because it is difficult to keep all the necessary equipment in proper operation. On the other hand, in these countries, such as Cuba, versatile equipment, such as LINAC, is preferred, which is not dedicated exclusively to SRS and further limits the possibilities of high casuistry. This fact, together with the particularities of the centers with limited resources, makes the analysis of patients treated with SRS in a context different from the usual one necessary.

The response to SRS is different in low-grade malignancy tumors than the response in high-grade lesions. In low-grade lesions, the absence of growth is the desired response, although, on many occasions, tumor changes such as necrosis, pseudoprogression, reduction of tumor volume, or changes in the edge of the tumor can be observed. In high-grade lesions (usually metastasis), the ideal response is complete disease regression or partial regression.

Surgery for skull base tumors is the treatment of choice, but when tumor control is not possible or surgery is contraindicated due to high neurological risks, SRS is a therapeutic option to consider. SRS in skull base tumors should be restricted to lesions that are at least 3 mm away from radiosensitive structures, such as the optic nerves and brainstem. Moreover, the doses in these cases are limited to the radiobiological tissue tolerance of these structures, which is usually in the order of 6-12 Gy.

The frequency of patients who had received previous radiation therapy was low because the tumors analyzed in this series are mostly histologically benign and rarely require radiation therapy as initial treatment. However, the highest incidence of previous surgery was seen in pontocerebellar angle tumors. This is due to the philosophy of preserving the function of the facial nerve in the surgery of these lesions, where a portion of the tumor capsule or the intra-meatal component is maintained to then be treated with SRS, as part of a combined treatment.

Radiosurgery in brain metastases

Although HRT has been used for decades in treating patients with brain metastases, its benefits have been questioned due to adverse effects (neurotoxicity), mainly dementia due to hypothalamic damage. On the other hand, local control is lower than in treatments with a higher dose than the target. The addition of SRS to HRT has increased local control and survival in patients with single brain metastases [32]. In patients with multiple brain metastases, SRS has improved local control but no additional benefit in survival over HRT alone. SRS also seems to be a viable alternative in patients operated on for brain metastases, as it radiates the tumor bed, which increases the local control of the lesions. There have been different clinical trials comparing SRS with HRT. Because there is class I evidence regarding equivalent survival among patients with one to three brain metastases, the omission of HRT in this group of patients has been widely accepted.

The maximum number of metastases to be treated with SRS is a controversial topic. The greatest current evidence is to treat patients with up to 10 metastases, although cases with 20 or more lesions have been treated. Reports from the University of Pittsburgh [33] suggest that treatment volume may be a more useful predictor than the number of metastases treated. The National Comprehensive Cancer Network recommends surgery in patients with more than three metastases. In this series, the largest number of lesions treated was two in a patient with two cerebellar metastases of breast adenocarcinoma. Patients with a greater number of metastases were not treated due to technical limitations of the equipment and because the INOR LINACs do not have teams working full-time to SRS. Only one day a week is available in the afternoon to perform the treatments, making it challenging to treat several lesions in a single patient, as it consumes additional time that is not available by the Department of Radiotherapy of the INOR. They are usually swamped in the treatment of other patients who require LINACs. Despite this, it can be affirmed that the results obtained with treating the two patients with metastases were favorable, with adequate local control during the follow-up studies.

Radiosurgery in meningiomas

Meningiomas are the most common benign tumors of the central nervous system (CNS). Among the treatment options are observation, SRS, and surgery. For decades, SRS was an alternative treatment to surgery for these lesions. Pollock BE et al. [34] treated 416 patients with SRS in a single session, with suggestive imaging or histologically confirmed lesions of low-grade meningioma (grade I). The mean dose in the margin was 16 Gy. Disease-free survival was 97% (considered excellent) at five years and 94% at 10 years. The frequency of complications at five years was only 11% [34]. SRS in grade II and III meningiomas have been less clear. Although the benefits of increased doses are controversial, in one of the most comprehensive reviews encompassing 647 grade II and III meningiomas treated with SRS, median margin doses of 16-22 Gy were found to be beneficial. [35]. In the present study, patients treated with meningioma had good local control, as the disease remained stable during the follow-up period.

Radiosurgery in vestibular schwannomas

Vestibular schwannomas are the second most common benign intracranial tumor after meningioma. The therapeutic options are the same as the latter. These lesions, mainly due to their location in a complex area and the need to reduce complications related to the function of the cranial nerves, constitute the most common benign tumor treated with SRS. To date, there are no studies with a high level of evidence comparing treatment with surgery to SRS. This may be because there are centers dedicated to one or another modality, so each center or surgeon selects the treatment option according to their possibilities and preferences. In this sense, INOR has the strength that surgery and SRS are performed by the same team, so the selection of the treatment option is based on the needs of the patient.

Local control of schwannomas treated with SRS is 90% at five years, with only 4% of patients requiring further treatment [18]. A high dose offers adequate local control but increases the chances of facial paralysis. On the other hand, a sub-therapeutic dose reduces the chances of facial paralysis but is associated with poor local control. The frequency of facial paralysis after SRS has been substantially reduced due to the reduction of the administered dose while maintaining therapeutic effectiveness. Currently, the recommended dose is 12-13 Gy. The preservation of useful hearing appears to be associated with a cochlear dose of less than 4 Gy. At a dose of 12-13 Gy, hearing conservation is about 70% at five years [36]. The lesions treated in the present series showed good local control without tumor pseudoprogression. However, transient facial paralysis was observed in one patient and permanent in another. On the other hand, the latter patient had a considerably larger lesion, for which it was necessary to raise the dose to the tumor margin, which increased the risk of facial paralysis.

Analysis of local control of treated lesions

This series shows good local control in all the treated lesions. These results coincide with what has been reported in the literature, with prevalence ranging from 34% to 100%. In vestibular schwannomas and meningiomas, tumor control can reach up to 100% because these tumors have a slow growth rate, and their stability can be misinterpreted as a response to treatment.

It has been described that tumor pseudoprogression can be observed in up to 30% of vestibular schwannomas during the first six months and up to the first year after SRS, and then remit at two years, so surgical treatment is rarely indicated, and patients are managed with narrow clinical-radiological follow-up and steroids [37]. However, surgical treatment may be considered in certain cases with significant clinical manifestations or life-threatening risks. There were no patients with pseudoprogression or tumor progression in the present series.

Analysis of associated complications

The frequency of acute complications in our series was low, and all those observed were minor. Edema associated with SRS is well known. In a study conducted by Harat M et al. [38] in a heterogeneous population of patients treated with SRS, edema was observed in 17% of cases. These authors found no association between the presence of edema and tumor size, dose escalation, or histopathological diagnosis. The absence of association between edema and histopathological diagnosis or size suggests a common etiology for the formation of post-SRS edema. In the present series, it was observed in three patients who only required steroid treatment and had clinical remission of symptoms.

It has been observed that edema increases during the first 6-9 months of SRS and begins to decrease after this period [38]. Not all cerebral edemas are symptomatic or require treatment. However, if cerebral edema is observed, close imaging and follow-up visits every three months are recommended, even in the absence of symptoms.

Limitations of radiosurgery

In patients with lesions larger than 3 cm or in proximity to critical structures and eloquent areas, some authors propose fractionation, which provides a solution because it allows preserving normal tissues through intact DNA repair mechanisms while producing lysis of tumor cells [2]. In this way, hypofractionated SRS provides the benefits of single-session SRS for tumor control while preserving adjacent structures, which is particularly desirable in treating skull base lesions.

Study limitations

The limitations of the present study include its retrospective nature, a biased selection of patients, and the small size of a series mixed with different lesions. However, most data on SRS are from retrospective studies of individual centers; few are prospective, and none of them are randomized controlled trials. On the other hand, these are the latest experiences of this treatment modality at the national level and the first experience in Cuba with the availability of two different SRS systems and outpatient treatment. A longer follow-up period is also necessary to comment on the safety and efficacy of the treatment.

## Conclusions

This retrospective study of patients treated for intracranial malignancies using SRS was predominated by extra-axial lesions. The treatment modality proved effective in the local control of treated lesions with a low complication rate. Most complications were minor such as perilesional edema. SRS can be carefully implemented on an outpatient basis on selected patients. Studies with a larger sample of patients and longer follow-up periods would allow for a better correlation of dosimetric variables to the clinical-imaging response.

## References

[1] Julie DA, Knisely JP (2020). Stereotactic radiosurgery technology. Central Nervous System Metastases.

[2] Beyzadeoglu M, Sager O, Dincoglan F (2020). Single fraction stereotactic radiosurgery (SRS) versus fractionated stereotactic radiotherapy (FSRT) for vestibular schwannoma (VS). J Surg Surgical Res.

[3] Gutiérrez-Aceves GA, Celis-Lopez MA, Garcia CP, Reyes-Moreno I, Gonzalez-Aguilar A, Rodríguez-Camacho A (2021). Radiosurgery for brain tumors. Principles of Neuro-Oncology.

[4] Gilbo P, Zhang I, Knisely J (2017). Stereotactic radiosurgery of the brain: a review of common indications. Chin Clin Oncol.

[5] Warsi NM, Karmur BS, Brar K (2020). The role of stereotactic radiosurgery in the management of brain metastases from a health-economic perspective: a systematic review. Neurosurgery.

[6] Lehrer EJ, Snyder MH, Desai BD (2020). Clinical and radiographic adverse events after Gamma Knife radiosurgery for brainstem lesions: a dosimetric analysis. Radiother Oncol.

[7] Gatterbauer B, Hirschmann D, Eberherr N (2020). Toxicity and efficacy of Gamma Knife radiosurgery for brain metastases in melanoma patients treated with immunotherapy or targeted therapy-A retrospective cohort study. Cancer Med.

[8] Ruggieri R, Naccarato S, Mazzola R, Ricchetti F, Corradini S, Fiorentino A, Alongi F (2018). Linac-based VMAT radiosurgery for multiple brain lesions: comparison between a conventional multi-isocenter approach and a new dedicated mono-isocenter technique. Radiat Oncol.

[9] Hanna SA, Mancini A, Dal Col AH, Asso RN, Neves-Junior WF (2019). Frameless image-guided radiosurgery for multiple brain metastasis using VMAT: a review and an institutional experience. Front Oncol.

[10] Alongi F, Fiorentino A, Gregucci F (2019). First experience and clinical results using a new non-coplanar mono-isocenter technique (HyperArc™) for Linac-based VMAT radiosurgery in brain metastases. J Cancer Res Clin Oncol.

[11] Hofmaier J, Bodensohn R, Garny S (2019). Single isocenter stereotactic radiosurgery for patients with multiple brain metastases: dosimetric comparison of VMAT and a dedicated DCAT planning tool. Radiat Oncol.

[12] (2021). WMA Declaration of Helsinki - Ethical Principles for Medical Research Involving Human Subjects. https://www.wma.net/policies-post/wma-declaration-of-helsinki-ethical-principles-for-medical-research-involving-human-subjects/.

[13] Levivier M, Wikier D, Goldman S (2000). Integration of the metabolic data of positron emission tomography in the dosimetry planning of radiosurgery with the gamma knife: early experience with brain tumors. J Neurosurg.

[14] Dindo D, Demartines N, Clavien PA (2004). Classification of surgical complications: a new proposal with evaluation in a cohort of 6336 patients and results of a survey. Ann Surg.

[15] Zhu S, Brodin NP, Garg MK, LaSala PA, Tomé WA (2021). Systematic review and meta-analysis of the dose-response and risk factors for obliteration of arteriovenous malformations following radiosurgery: an update based on the last 20 years of published clinical evidence. Neurosurgery Open.

[16] Kim BS, Kim KH, Lee MH, Lee JI (2019). Stereotactic radiosurgery for brainstem cavernous malformations: an updated systematic review and meta-analysis. World Neurosurg.

[17] Alatriste-Martínez S, Moreno-Jiménez S, Gutiérrez-Aceves GA, Suárez-Campos JJ, García-Garduño OA, Rosas-Cabral A, Celis-López MÁ (2019). Linear accelerator-based radiosurgery of grade I intracranial meningiomas. World Neurosurg X.

[18] Alam S, Chaurasia BK, Shalike N (2018). Surgical management of clinoidal meningiomas: 10 cases analysis. Neuroimmunol Neuroinflammation.

[19] Anselmo P, Casale M, Arcidiacono F (2020). Twelve-year results of LINAC-based radiosurgery for vestibular schwannomas. Strahlenther Onkol.

[20] Zhang L, Chen W, Ding C, Hu Y, Tian Y, Luo H, Chen J (2021). Gamma Knife radiosurgery as the initial treatment for elderly patients with nonfunctioning pituitary adenomas. J Neurooncol.

[21] Rahman A, Ahmed N, Baniya P, Scalia G, Umana GE, Chaurasia B (2020). Primary sellar neuroblastoma mimicking invasive pituitary adenoma: a systematic review. J Neurosurg Sci.

[22] Lior U, Rotem H, Uzi N, Roberto S (2020). LINAC radiosurgery for glomus jugulare tumors: retrospective - cohort study of 23 patients. Acta Neurochir (Wien).

[23] Deora H, Tripathi M, Tewari MK, Ahuja CK, Kumar N, Kaur A, Kamboj P (2020). Role of gamma knife radiosurgery in the management of intracranial gliomas. Neurol India.

[24] Gupta A, Yadav BS, Ballari N, Das N, Robert N (2021). LINAC-based stereotactic radiosurgery/radiotherapy for brain metastases in patients with breast cancer. J Radiother Prac.

[25] Cahill J, Ibrahim R, Mezey G (2021). Gamma Knife stereotactic radiosurgery for the treatment of chordomas and chondrosarcomas. Acta Neurochir (Wien).

[26] Zhao M, Fu X, Zhang Z, Ma L, Wang X, Li X (2021). Gamma Knife radiosurgery for high-grade gliomas: single-center experience of six years in China. Stereotact Funct Neurosurg.

[27] Abdali A, Kalinin PL, Trunin YY (2021). CyberKnife for the management of Cushing's disease: our institutional experience and review of literature. Br J Neurosurg.

[28] Abdali A, Astaf Eva L, Trunin Y (2020). Modern methods of stereotactic radiosurgery and radiotherapy for the treatment of Cushing disease. Neurol India.

[29] Sachdev S, Sita TL, Shlobin NA, Gopalakrishnan M, Sucholeiki R, Régis J, Bandt SK (2020). Completion corpus callosotomy with stereotactic radiosurgery for drug-resistant, intractable epilepsy. World Neurosurg.

[30] Park SH, Chang JW (2020). Gamma Knife radiosurgery on the trigeminal root entry zone for idiopathic trigeminal neuralgia: results and a review of the literature. Yonsei Med J.

[31] Higuchi Y, Matsuda S, Serizawa T (2017). Gamma knife radiosurgery in movement disorders: indications and limitations. Mov Disord.

[32] Bilger A, Frenzel F, Oehlke O (2017). Local control and overall survival after frameless radiosurgery: a single center experience. Clin Transl Radiat Oncol.

[33] Niranjan A, Monaco E, Flickinger J, Lunsford LD (2019). Guidelines for multiple brain metastases radiosurgery. Prog Neurol Surg.

[34] Pollock BE, Stafford SL, Link MJ (2013). Stereotactic radiosurgery of intracranial meningiomas. Neurosurg Clin N Am.

[35] Ding D, Starke RM, Hantzmon J, Yen CP, Williams BJ, Sheehan JP (2013). The role of radiosurgery in the management of WHO Grade II and III intracranial meningiomas. Neurosurg Focus.

[36] Chung LK, Ung N, Sheppard JP (2018). Impact of cochlear dose on hearing preservation following stereotactic radiosurgery and fractionated stereotactic radiotherapy for the treatment of vestibular schwannoma. J Neurol Surg B Skull Base.

[37] Yang HC, Wu CC, Lee CC (2021). Prediction of pseudoprogression and long-term outcome of vestibular schwannoma after Gamma Knife radiosurgery based on preradiosurgical MR radiomics. Radiother Oncol.

[38] Harat M, Lebioda A, Lasota J, Makarewicz R (2017). Evaluation of brain edema formation defined by MRI after LINAC-based stereotactic radiosurgery. Radiol Oncol.

